# Acute Kidney Injury Caused by Levetiracetam in a Patient With Status Epilepticus

**DOI:** 10.7759/cureus.8814

**Published:** 2020-06-24

**Authors:** Burak Erdinc, Snigdha Ghanta, Alexander Andreev, Karim O Elkholy, Sonu Sahni

**Affiliations:** 1 Internal Medicine, Brookdale University Hospital Medical Center, Brooklyn, USA; 2 Research Medicine, New York Institute of Technology College of Osteopathic Medicine, New York, USA; 3 Primary Care, Touro College of Osteopathic Medicine, New York, USA

**Keywords:** levetiracetam, acute kidney injury, status epilepticus

## Abstract

Levetiracetam is a widely used, effective and usually well-tolerated anti-epileptic medicine. It is mostly excreted by kidneys and requires dose adjustment according to the glomerular filtration rate. Very few case reports have been published in the literature about levetiracetam causing acute kidney injury (AKI). We present a case of a 26-year-old male with a seizure disorder on levetiracetam, presented with status epilepticus requiring intubation for airway protection. He received 4 g of intravenous levetiracetam as a loading dose and continued with a maintenance dose of 750 mg intravenous every 12 hours. He had signs of AKI on day two and creatinine eventually reached a maximum level of 12.2 mg/dL. His kidney function improved to his new baseline in a period of 30 days without requiring renal replacement therapy. He did not have significant rhabdomyolysis and his kidney function started improving right after his anti-epileptic therapy was switched to valproic acid pointing towards levetiracetam as the primary cause of kidney injury. Clinicians should be aware that levetiracetam can cause AKI on patients with a seizure disorder, especially when administered in high doses. Kidney function should be monitored closely and patients should be treated aggressively with intravenous fluids when they have any signs of rhabdomyolysis to prevent further kidney damage.

## Introduction

Levetiracetam is currently one of the most commonly used anti-epileptic drugs (AEDs). Due to its mechanism of action not binding it to plasma proteins and not being dependent on the cytochrome p450 system, its pharmacokinetic interactions are minimal. Primary elimination of levetiracetam occurs through renal excretion and dose adjustments are necessary for patients with moderate to severe kidney impairment [1-3]⁠. The most commonly reported side effects of levetiracetam are somnolence, asthenia, dizziness, headache, and rarely behavioral adverse effects [4]⁠. Despite its mechanism of action and renal excretion, there has been scant literature of acute kidney injury (AKI) secondary to levetiracetam use [5-8]⁠. On the other hand, a retrospective population-based cohort study involving 3,980 patients on levetiracetam treatment reported no significant risk of developing AKI within 180 days [9]⁠. Herein we report a case of AKI caused by levetiracetam, which was administered with a high loading dose in a critical unit setting.

## Case presentation

A 26-year-old Hispanic male with a past medical history only significant for epilepsy was witnessed by family members to have five episodes of tonic-clonic seizures without regaining consciousness between seizure episodes. As per emergency medical services (EMS) reported, the patient was found lying on the floor with fecal and urinary incontinence. Initial vital signs by EMS showed a blood pressure (BP) of 183/108 mmHg, heart rate (HR) of 103 beats per minute (bpm), and blood glucose of 302 mg/dL. He was able to mumble some answer verbally but remained disoriented. As the patient was being transferred to the ambulance, he became aggressive and combative. He was administered 10 mg midazolam intramuscularly and brought in the ED at Brookdale University Hospital Medicine Center. Prior to the presentation, home medications included levetiracetam 750 mg tablets twice daily with which he was not fully compliant as per his family. Chart review revealed multiple ED visits for seizures due to non-compliance with the medication. In the ED, he received another dose of midazolam, 4 mg to terminate the residual seizure activity and also received haloperidol and ketamine due to agitation with combativeness after his initial seizure episode. Vital signs in the ED were within normal limits except a HR of 142 bpm. Electrocardiogram showed sinus tachycardia. The patient remained afebrile, but it was noted that his BP began to rise and was recorded at 133/109 mmHg. Soon thereafter, the patient was noticed to be in a phase of status epilepticus and eventually was intubated for airway protection.

Physical examination revealed the patient to be intubated and sedated, equal-sized pupils reactive to light, intact brainstem reflexes, no signs of major trauma on his body. He received 4 g of intravenous levetiracetam as a loading dose and was continued with 1000 mg of levetiracetam intravenously every 12 hours thereafter. Initial laboratory investigations revealed an elevated lactic acid level of >12 mmol/L, creatinine level of 0.9 mg/dL and creatinine kinase level of 1004 U/L. Clinical laboratory data has been shown in Table 1. His baseline creatinine level was within normal limits at 0.9 mg/dL about four years ago and he did not have any previous history of kidney disease. Venous blood gas showed a pH of 7.07 and bicarbonate level of 14.4 mmol/L indicating metabolic acidosis most likely due to prolonged seizure activity. Urine toxicology was positive for cannabinoids. CT imaging of the head was negative for acute pathology.

**Table 1:** Initial laboratory investigations

Test	Reference Range	Result
White Blood Cell Count	4.10 - 10.10 10x3/uL	25.90 (H)
Hemoglobin	12.9 - 16.7 g/dL	14.8
Platelets	153 - 328 10x3/uL	416 (H)
Neutrophils, Absolute	1.40 - 6.80 10x3/uL	22.50 (H)
Lymphocytes, Absolute	1.10 - 2.90 10x3/uL	2.6
Monocytes, Absolute	0.20 - 1.00 10x3/uL	0.7
Eosinophils, Absolute	0.00 - 0.40 10x3/uL	0.1
Basophils, Absolute	0.00 - 0.10 10x3/uL	0.1
Glucose	74 - 106 mg/dL	145 (H)
Blood Urea Nitrogen	9.0 - 20.0 mg/dL	16
Creatinine	0.66 - 1.25 mg/dL	1.22
Sodium	133 - 145 mEq/L	137
Potassium	3.5 - 5.1 mEq/L	4.5
Chloride	98 - 107 mEq/L	109 (H)
Bicarbonate	22 - 30 mEq/L	19 (L)
Calcium	8.4 - 10.2 mg/dL	10.8 (H)
Anion Gap	8-12 mEq/L	9
Protein, Total	6.3 - 8.2 g/dL	9.5 (H)
Albumin	3.5 - 5.0 g/dL	5.3 (H)
Bilirubin, Total	0.2 - 1.3 mg/dL	1
Alanine transaminase (ALT)	21 - 72 U/L	72
Aspartate transaminase (AST)	17 - 59 U/L	43
Lactate	0.70 - 2.10 mmol/L	>12 (H)
Creatinine Kinase (CPK)	55 - 170 U/L	1004 (H)
Urine sodium	30.0 - 90.0 mEq/L	96.0 (H)
Urine potassium	mEq/L	22.2
Urine protein	5.0 - 11.0 mg/dL	95.0 (H)
Urine creatinine	mg/dL	127.2
Urine urea nitrogen	mg/dL	87

The patient was transferred to the intensive care unit and repeat laboratory testing the next day showed blood urea nitrogen of 17 mg/dL and significant elevation in creatinine level to 3.27 mg/dL without a significant increase in creatinine kinase 1377 U/L (reference value: 55-170 U/L). Urine electrolytes were obtained to investigate the acute rise in creatinine (Table 1) and FeNa (fraction excretion of sodium) was calculated at 8%. Initial urinalysis showed large blood but only 0-3 red blood cells on high power field microscopy, The patient also had iso-osmotic urine with a specific gravity of 1.010 and high urine sodium (96 mEq/L). He did not receive any intravenous contrast, had any other medications with known nephrotoxicity or had any episodes of hypotension, which might compromise kidney perfusion and cause AKI. 

**Figure 1:**
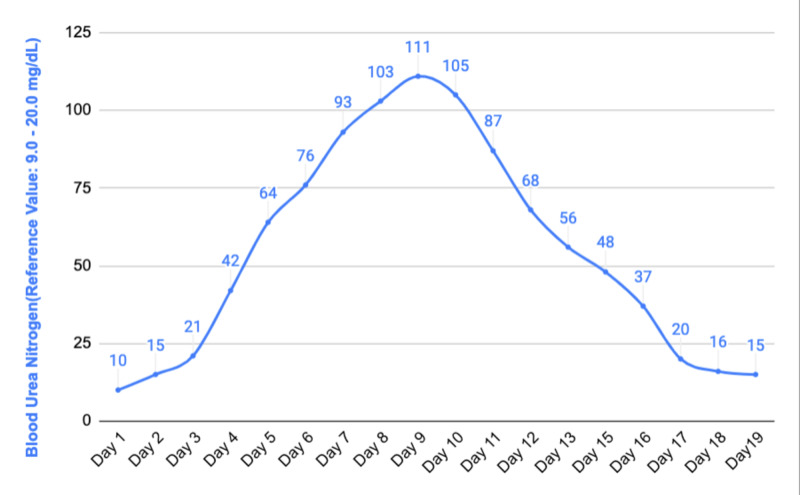
Blood urea nitrogen trend

**Figure 2:**
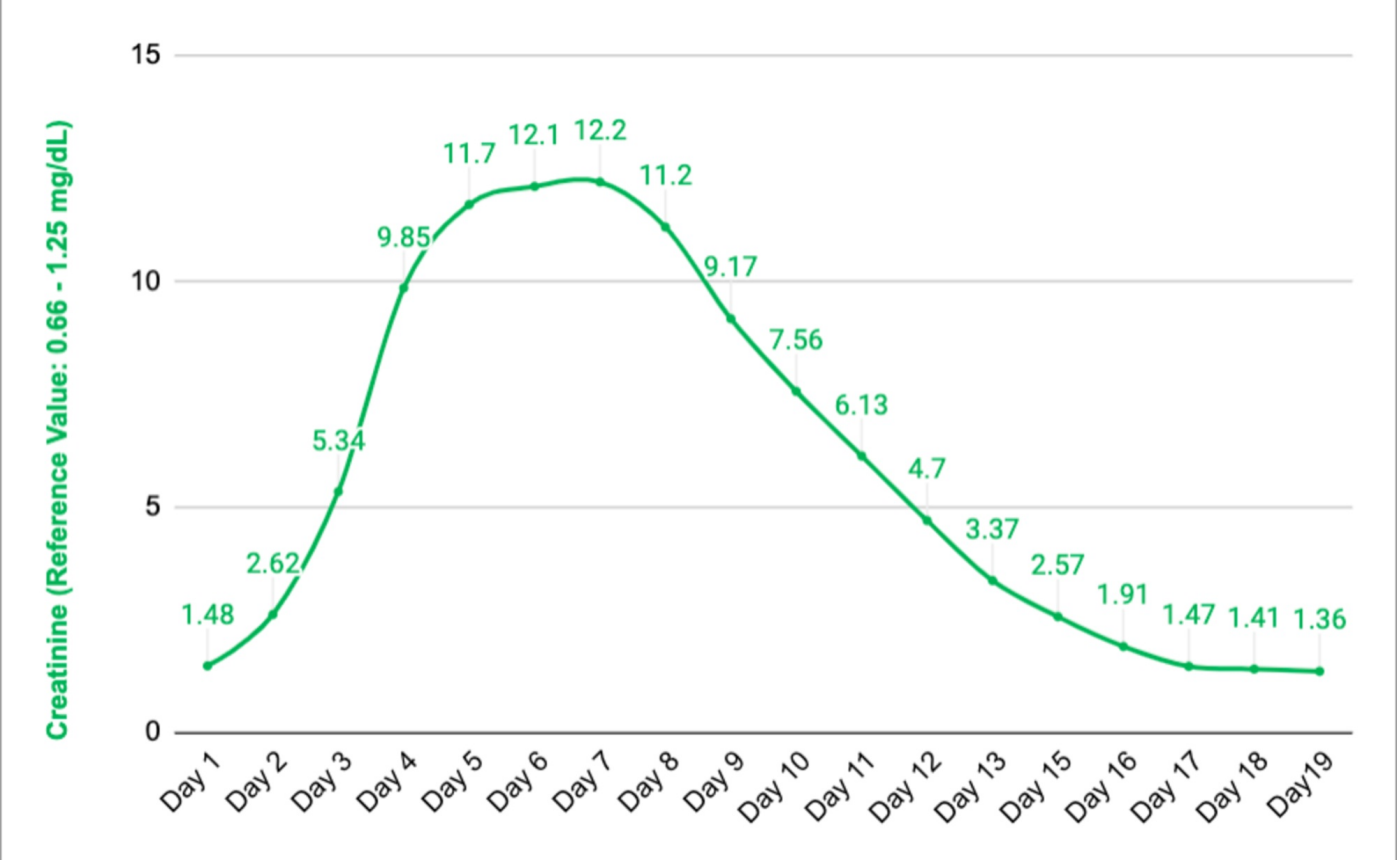
Creatinine trend

**Figure 3:**
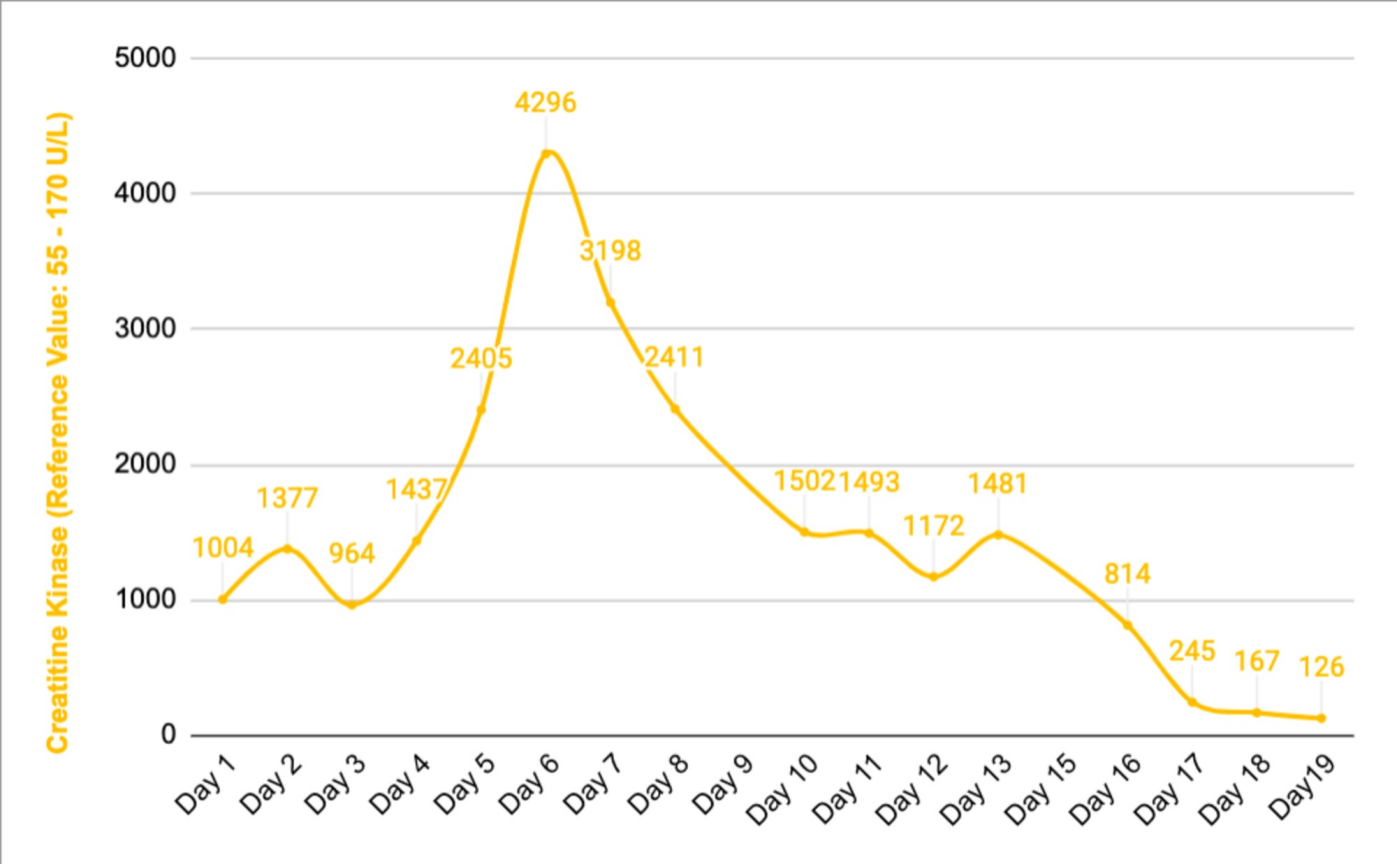
Creatinine kinase trend

The patient was assessed by a neurologist and valproic acid 500 mg twice daily was added to the anti-epileptic regimen to control seizures and levetiracetam was stopped since it was thought to be the culprit for AKI. Urine output was noted to decrease to 60 cc per hour and then the patient entered an oliguric phase. The patient received intravenous furosemide to keep him in non-oliguric and also received half normal saline interchangeably with 5% dextrose in water for volume expansion. The patient soon began to undergo brisk diuresis (up to 4L of urine per day) in the recovery phase of acute tubular necrosis (ATN). Creatinine levels continued trending down. Ultrasound of the retro-peritoneal organs showed normal kidneys without any signs of hydronephrosis. The patient’s urine output was monitored closely and he received large amounts of intravenous fluids in the recovery phase. His creatinine level came back to its new baseline level of 1.36 mg/dL after 20 days without requiring renal replacement therapy. Blood urea nitrogen, creatinine, and CPK trends have been shown in Figures 1-3, respectively. The patient was extubated successfully after 12 days, remained seizure-free and discharged home with outpatient follow-up. The patient has remained seizure-free and renal function has remained stable 30 days after discharge.

## Discussion

Due to its favorable safety profile, levetiracetam is a widely used and well-tolerated medication for different types of seizure disorders with minimal side effects. However, due to its renal excretion, there is a possibility of renal side effects and there remains a paucity in the literature about levetiracetam causing AKI. Our case demonstrates that levetiracetam may cause AKI, especially when it is administered with high loading doses.

Levetiracetam is a highly effective anti-epileptic medication with a high therapeutic index and good bioavailability. It minimally binds to proteins in the blood and reaches steady-state concentrations rapidly. Its interaction with liver-metabolized medications is limited since it is not dependent on the cytochrome p450 enzyme system. Levetiracetam is mostly (60%) excreted in the urine with no modifications and a fraction (24%) of it gets inactivated in the circulatory system before being excreted in the urine. Due to primarily being excreted in the urine, its dose should be adjusted according to creatinine clearance [1]⁠.

The most common adverse reactions secondary to levetiracetam use are fatigue, dizziness, headache and somnolence [4,10,11]⁠. In a large, open-label, community study on safety and efficacy of levetiracetam, 1,030 patients being treated for partial-onset seizures showed no evidence of kidney injury [4]⁠. In another open label, multi-center, single-arm study on 99 patients who were treated with 1000-3000 mg/day levetiracetam for refractory partial-onset seizures, patients' blood chemistry was followed up while on therapy. They reported no significant changes in patient’s blood urea nitrogen and creatinine levels, except they found blood in the urine of 10 patients, of whom nine were women who were perimenstrual [11].

Moreover, levetiracetam induced kidney injury has been reported in very few cases. In a case of a previously healthy 23-year-old woman who presented with new-onset seizures and was started levetiracetam 500 mg twice daily, on day two of therapy she had a rise in her blood urea nitrogen (20 mg/dL) and creatinine (2.48 mg/dL) levels from a normal baseline. Levetiracetam therapy was stopped and she started taking phenytoin for seizure prevention. Her kidney function improved to her baseline on follow-up physical examination two months after discharge [5]⁠. Another case of a 17-year-old female who was started on levetiracetam (250 mg twice daily) for new-onset partial complex seizures, presented to a local ED 10 days later with gastrointestinal side effects. She was found to have elevated creatinine of 3.3 mg/dL from a normal baseline and also had +3 blood and +3 protein in her dipstick urine test. Subsequent kidney biopsy confirmed the diagnosis of interstitial nephritis. Her kidney function recovered completely after 10 days of initiating oral prednisone treatment and switching levetiracetam to oxcarbazepine for seizure prevention [7]⁠. A third case of a 16-year-old, previously healthy male, who presented with generalized tonic-clonic seizures and was started levetiracetam therapy (750 mg twice daily) had a rise in his creatinine level (2.2 mg/dL) on day two from a normal baseline. The patient also complained of muscle pain and labs were significant for rhabdomyolysis with peak creatinine kinase level of 15,111 U/l, primarily contributing to AKI. Levetiracetam therapy was stopped on day five of his treatment and the patient was switched to divalproex sodium therapy for seizure prevention and received intravenous fluid resuscitation. His creatinine level normalized on day seven and the patient was safely discharged home [8]. A fourth case of a previously healthy 45-year-old male who started taking levetiracetam (starting dose was 500 mg twice daily which later gradually increased to 3000 mg/day) for seizure prevention due to newly discovered astrocytoma. He experienced progressively worsening gastrointestinal symptoms (mainly intractable nausea) and had an increased serum creatinine to 3.59 mg/dL from a normal baseline. Levetiracetam was the only medication he received during over the two month period and his fractional excretion of sodium (FeNa) was calculated more than 1%, indicating intrinsic causes of kidney injury rather than pre-renal AKI from dehydration. Levetiracetam was tapered off and he started taking lacosamide for seizure prevention. His creatinine level normalized to 1.34 mg/dL over one month [6].

## Conclusions

To summarize, levetiracetam can cause AKI on patients with a seizure disorder, more commonly when used in high doses as was seen in our case. In addition, rhabdomyolysis might contribute to kidney injury in patients with prolonged seizure activity. In these circumstances, levetiracetam therapy should be stopped immediately and patients should continue their treatment with an alternative anti-epileptic medication for seizure prevention, along with intravenous fluid resuscitation and motorization of kidney function. Kidney function usually recovers in a period of seven days to 30 days with appropriate therapy and follow-up according to previously reported case reports.
